# Supplementary material for: Glucocorticoid Receptor Alpha Targets SLC2A4 to Regulate Protein Synthesis and Breakdown in Porcine Skeletal Muscle Cells
Source: Biomolecules. 2021 May 12;11(5):721. doi: 10.3390/biom11050721 (PMC8151344; doi:10.3390/biom11050721)
Supplement: Supplementary file 1 [file biomolecules-11-00721-s001.zip › biomolecules-1091176-supplementary.pdf]

Supplementary Materials:

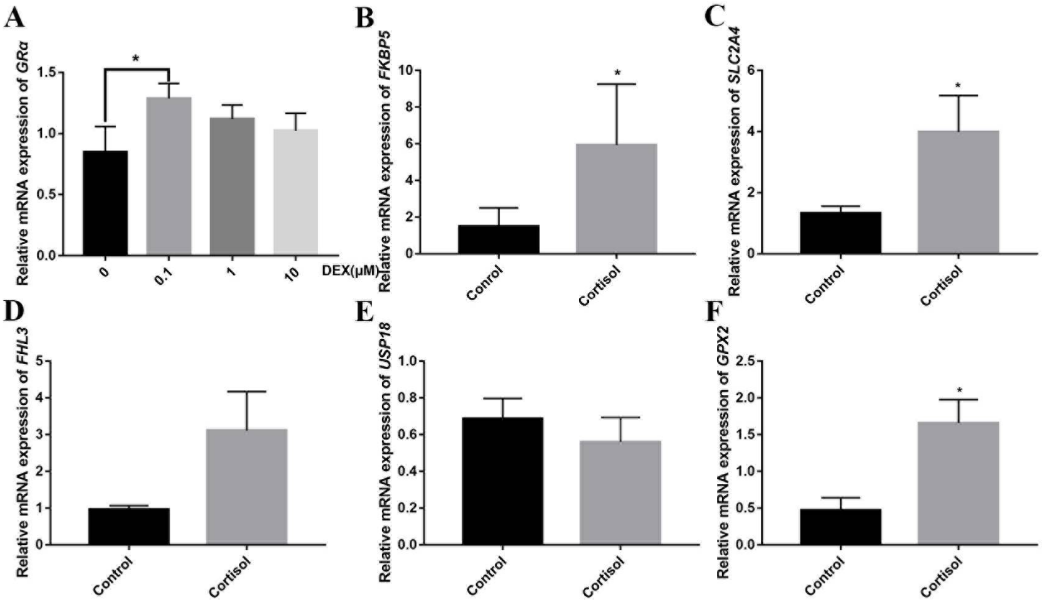

Figure S1. The optimal concentration of DEX in PSC cells and quantitative detection of differentially expressed genes in muscle. A: The mRNA expression of glucocorticoid receptor gene GR*α* at different DEX concentrations (0, 0.1, 1, 10 μM); B-F: The mRNA expression levels of *FKBP5, SLC2A4, FHL3, USP18* and *GPX2* in longissimus dorsi muscle of the cortisol group (n=6) and the control group(n=6), respectively. * indicate significant difference at P<0.05.

Promoter prediction for 1 eukaryotic sequence sequence with score cutoff 0.80(transcription start shown in larger font):

Promoter predictions for 1:

Start	End	Score	Promoter Sequence
1623	1673	1.00	CGTAGGAGCTAAAAATAACCGCCGTGGGTTACTCTAGGCCATTGGCCCGC
2042	2092	0.89	TGGGTGCTGTGAAAGGCGTGGCCCTGGGGGCTGCGGGGGCTGCTCCTCGG

GAGGCCCGTTTTCCCAGCCGCGTGCGCTCCCCTACCCAGTGTTCTGCTGCTCTCGGGACATGCCAACTCTAGGTCGTGCCCTCCTCCCTGGGCAGGAT

TTAGCTGGGGATTCAAACCCAGAGCGGCCTCCAGGGAGCGTGTCCCAAGTCGTCCACGCGGCTAGCACATTCTTGGCAACTCAGGGGCCTCAGGAC

TGGTGACGTGGTTGGTGTGACCACCTGTCCCTTGGGCCGCTTCCAGTAACCAACGTGGGCCACGGGTGTAGGGGAAGGACCAGATAGGCAGAGCCC

GGAGCAGGGATGTACGGAAAGAGGACCACCAAGTCCAGACACCCGACCCGCTAGGAACGGAATTTCCTGTCCCCGGGGCCATCACCGCGTGGGGA

GCGTGTCGCGGACCCTTTAAGGCTCCATCTCCCTGCCACCACCACCCCGCCCGGGACAGGCCAGGACACCGGGGACCTGACATTTGGCGTCTCCCA

A**CGTAGGAGCTAAAAATAACCGCCGTGGGTTACTCTAGGCCATTGGCCCGC**ACCCACCCCGCGCGCCGCCTCGCGCTGTCTCGGGCCGCCCGCG

Predicted sequence with promoter active site 1

GTCTCAACCGGGCAAGTGTACCTGGCCCATCTCCTCTCAGACCCCGCGGTTCCGAGCCAACGCGCAGGGCTTTGAAGTCGCTTCTCCGGCCCCCAAG

CTTGGCAAGTGGGCGGAGTCTTCGCACTTAAGGCGACGGAGCCGCCAGGTCTCCCAGGACCCTTTTCTCAAACACTTCCCGGCGGAAAGCTGGCGG

GAACCGAGGGGCGTGTCTCGCCGGGCACGCTAAGGGGCGTGGCCTCGCCGGCCAACCCGACGGCTGGTGCCAAATTCCCAGCCCGAGCTCAGGGA

GGGGCGTGGCCTTCTGGGGTGTGCGGGCTCCTGGCCAA**TGGGTGCTGTGAAAGGCGTGGCCCTGGGGGCTGCGGGGGCTGCTCCTCGG**GTCTTTT

Predicted sequence with promoter active site2

CCCCCCAAGCCAGGCTCCACCAGATCCCTGGGAGCTACACTCTGGCTCCTCGGCTTGTGGCTGTGGGTCCGACCCGGCCCGCCCTCCGTACTGCGCT

TCGGGACCCCCGTCGCCCCTGTGTCCTTCTGCCCGCCAGGCTGGGGCCACAGACCCCCAGTCGCCTCTCTCCGGTCTCCGGCAGCTCCTGCGCCTCC

AGCTCCTAAGACAAG**ATG**CCGTCGGGCTTCCAACAGATCGGCTCCGAA

Translation start site
